# Prenatal Paracetamol Exposure and Wheezing in Childhood: Causation or Confounding?

**DOI:** 10.1371/journal.pone.0135775

**Published:** 2015-08-25

**Authors:** Enrica Migliore, Daniela Zugna, Claudia Galassi, Franco Merletti, Luigi Gagliardi, Laura Rasero, Morena Trevisan, Franca Rusconi, Lorenzo Richiardi

**Affiliations:** 1 Cancer Epidemiology Unit, Department of Medical Sciences, AOU Città della Salute e della Scienza di Torino, CPO Piemonte, CERMS, and University of Turin, Turin, Italy; 2 Woman and Child Health Department, Pediatrics and Neonatology Division, Ospedale Versilia, Viareggio, Italy; 3 Department of Experimental and Clinical Medicine, University of Florence, Florence, Italy; 4 Unit of Epidemiology, ‘Anna Meyer’ Children’s University Hospital, Florence, Italy; University of Oxford, UNITED KINGDOM

## Abstract

**Background:**

Several studies have reported an increased risk of wheezing in the children of mothers who used paracetamol during pregnancy. We evaluated to what extent this association is explained by confounding.

**Methods:**

We investigated the association between maternal paracetamol use in the first and third trimester of pregnancy and ever wheezing or recurrent wheezing/asthma in infants in the NINFEA cohort study. Risks ratios (RR) and 95% confidence intervals (CI) were estimated after adjustment for confounders, including maternal infections and antibiotic use during pregnancy.

**Results:**

The prevalence of maternal paracetamol use was 30.6% during the first and 36.7% during the third trimester of pregnancy. The prevalence of ever wheezing and recurrent wheezing/asthma was 16.9% and 5.6%, respectively. After full adjustment, the RR for ever wheezing decreased from 1.25 [1.07–1.47] to 1.10 [0.94–1.30] in the first, and from 1.26 [1.08–1.47] to 1.10 [0.93–1.29] in the third trimester. A similar pattern was observed for recurrent wheezing/asthma. Duration of maternal paracetamol use was not associated with either outcome. Further analyses on paracetamol use for three non-infectious disorders (sciatica, migraine, and headache) revealed no increased risk of wheezing in children.

**Conclusion:**

The association between maternal paracetamol use during pregnancy and infant wheezing is mainly, if not completely explained by confounding.

## Introduction

Paracetamol is one of the most commonly used medications in pregnant women and has almost completely replaced aspirin as an analgesic and antipyretic in children. In late 1980s, some authors suggested that this increase in paracetamol use might contribute to an increase in the prevalence of asthma, particularly in the paediatric population [1].

An association between paracetamol exposure in early childhood and the occurrence of asthma in school-age children has been reported and reviewed repeatedly [2–4]. However, the observational and frequently retrospective design of the majority of these studies did not allow firm conclusions to be drawn regarding the causality of the observed association. The most recent evidence [2, 3, 5] suggests that the association between paracetamol exposure in early childhood and paediatric asthma may be at least partly due to different types of bias, particularly confounding by indication. Indeed, paracetamol is used to treat several disorders in children, the most important being respiratory tract infections, which are also associated with wheezing and asthma development [6]. In fact, Cheelo et al. [3] observed that the association between paracetamol exposure in early childhood and subsequent paediatric asthma decreased when the data were also adjusted for early respiratory tract infections. This finding was confirmed in the recent study by Sordillo et al. [5]. Furthermore, there were two studies in the review by Cheelo et al. [3] that examined the effects of paracetamol given to children for non-respiratory infections [6, 7], and neither found an increased risk of asthma associated with paracetamol use.

Some cohort studies have reported an association between maternal paracetamol use during pregnancy and risk of wheezing or asthma in children [3, 5, 8, 9]. However, as this evidence is also based on observational studies it is still unclear whether the association between maternal paracetamol use in pregnancy and wheezing or asthma in children is causal or at least partly spurious [3, 4, 8]. Maternal paracetamol use could be a proxy for maternal disorders that are associated with childhood asthma, such as respiratory infections [10]. Furthermore, wheezing in childhood, particularly in early childhood, is often triggered by infections. Therefore, in addition to the infections themselves, any condition that favours or is associated with maternal infections (e.g. maternal smoking, maternal antibiotic use, siblings) could be related to an increased risk of infection in children and thus to wheezing in children who are predisposed. It should also be noted that the association between paracetamol use during pregnancy and asthma in school-aged children has not been supported in some studies [5, 11–14].

We used data from the NINFEA (Nascita e INFanzia: gli Effetti dell'Ambiente) cohort study, an Italian birth cohort study, to assess to what extent the potential association between maternal paracetamol use and ever or recurrent infant wheezing/asthma is explained by confounding.

## Materials and Methods

The NINFEA cohort study is an ongoing web-based birth cohort. It was started in 2005 in the city of Turin and was progressively extended to the rest of Italy, with the aim of investigating the effects of early-life exposures on the health of newborns, adolescents, and adults (www.progettoninfea.it) [15]. Cohort members are children of mothers who have access to the internet and have enough knowledge of the Italian language to complete online questionnaires. The existence of the study is actively publicised in hospitals and obstetrical clinics, with the collaboration of health personnel who distribute leaflets and posters; it is also passively publicised on the internet via links and discussion forums. Approximately 75% of participants are recruited through active methods, 20% through passive methods, and 5% through both methods. Women who are interested in participating in the study complete a first online questionnaire (baseline questionnaire) at any time during their pregnancy. This is followed by four follow-up online questionnaires, which are completed by the mothers when their child turns 6 months, 18 months, 4 years, and 7 years of age, respectively. Members of the NINFEA cohort study originate from a selected population, but baseline selection does not imply biased associational estimates in cohort studies [16–18]. The NINFEA cohort study was approved by the Ethical Committee of the San Giovanni Battista Hospital-CTO/CRF/Maria Adelaide Hospital of Turin. Online written informed consent was obtained from all the mothers of the study participants at the time of completing the first questionnaire, in accordance with IRB approval.

Data were obtained from the NINFEA database version 2013.03. Children with available information on the outcome and exposure of interest, and whose mothers had filled in both baseline questionnaire, and the 6-month and the 18-month questionnaire (response proportion for 18-month questionnaire: 84%) at the time of data download were eligible for inclusion. Children born to mothers who had filled in the baseline questionnaire before the end of their first trimester of pregnancy were excluded due to incomplete information on the first trimester.

### Exposure data

Information on maternal paracetamol use and duration of paracetamol use (number of days) was obtained from the baseline questionnaire for the first trimester of pregnancy and from the 6-month questionnaire for the third trimester of pregnancy (S1 Table).

We also evaluated maternal paracetamol use during pregnancy for three non-infectious disorders that are frequently treated with paracetamol: sciatica, headache, and migraine (S2 Table). These disorders are included in a pre-defined checklist of diseases in the baseline questionnaire; women were asked to report if they had ever been diagnosed with these disorders by a doctor, and if so the drugs they used to treat them in the first and third trimester of pregnancy.

We did not consider maternal paracetamol use in the second trimester of pregnancy due to a lack of information. Indeed, as women could complete the baseline questionnaire at any time during their pregnancy, those who completed the baseline questionnaire before their 6^th^ month of pregnancy had complete information on the first trimester but not on the second trimester.

Information on duration of paracetamol use was collected in the questionnaire using pre-specified categories (1–2 days, 3–7 days and more than 7 days per month) until 2008. Thereafter, information was collected using the actual number of days. We harmonised the two versions of the questionnaire by converting the categorical variable into the corresponding stratum-specific average number of days.

### Outcome variables

Information on the outcomes ever wheezing and recurrent wheezing/asthma was obtained from the 6-month and 18-month questionnaires, which used questions derived from the ISAAC questionnaires (S3 Table) [19]. Ever wheezing was defined as at least one episode of wheezing or whistling in the chest in the first 6 months of life, or between 6 and 18 months of life. Recurrent wheezing/asthma was defined as wheezing reported in both the 6-month and 18-month questionnaires or asthma diagnosed by a doctor in either of these questionnaires.

### Statistical analyses

Crude risks ratios (RR_crude_) and adjusted RR (RR_adjusted_) were estimated using poisson regression models, separately for maternal paracetamol use in the first and third trimester of pregnancy. Models were first adjusted for several maternal characteristics, conditions, and disorders that are potentially associated with infant wheezing and possibly associated with paracetamol use during pregnancy (educational level, age at delivery, smoking during pregnancy, siblings, asthma/asthmatic bronchitis, and maternal allergic rhinitis/hay fever) [3, 20]. Models were then further adjusted for maternal respiratory tract infections in the first or third trimester of pregnancy (otitis/sinusitis/ throat infection, bronchitis or flu, and cold) and for factors associated with maternal infections (fever >38°C and antibiotic use during pregnancy) [2, 3]. The rationale for this additional adjustment was that maternal infection may be associated with the risk of respiratory diseases in children and may also indicate maternal paracetamol use.

We also modelled paracetamol use for the three non-infectious maternal disorders (sciatica, headache, and migraine) separately in the first and third trimester of pregnancy in association with ever wheezing and recurrent wheezing/asthma. Specifically, we compared mothers who used paracetamol to treat any of these disorders with mothers affected by these disorders who did not use paracetamol to treat their symptoms. These non-infectious disorders were selected because they are not risk factors for wheezing or asthma; as such they can be used to discriminate between mothers who did and did not use paracetamol for disorders that are unrelated to the risk of wheezing or asthma in children, by assuming paracetamol use for other diseases is equally distributed in each category. This analysis was adjusted only for the first set of confounders.

Since women could contribute more than one pregnancy to the cohort, we estimated robust variance using clustered sandwich estimators to allow for intra-group correlation. All regression models were performed on the set of children with no missing data for any of the outcome, exposure, or confounding variables.

The relationship between wheezing and duration of the paracetamol use (expressed as increment of risk per day of use) in the first and third trimester was further studied by a restricted cubic spline regression of days of use to test whether there was a non-linear dose-response shape. In a sensitivity analysis, we evaluated the robustness of our results using a propensity score-based analysis.[21] Specifically, the probability of paracetamol use was modelled conditioning on potential determinants of paracetamol use, such as maternal allergic rhinitis, asthma and asthmatic bronchitis, maternal disorders in the first/third trimester of pregnancy which may or not may be treated with paracetamol, and maternal antibiotic use. After checking for the absence of subjects outside the region of common support of the propensity score, the score was introduced in the poisson regression model for the outcome variables, further adjusting for maternal age at delivery, the maternal characteristics educational level, age at delivery, smoking during pregnancy, and parity.

All analyses concerning the third trimester were further adjusted for gestational duration, as, keeping all other things the same, the probability of taking any drug is, by definition, higher in a longer than a shorter pregnancy. Analyses were performed using statistical software STATA 12.1.

## Results

There were 4254 children born to mothers who filled in the 18-month questionnaire. We excluded 144 because they were twins, and 572 because the baseline questionnaire was completed before the end of the first trimester of pregnancy. Of the remaining 3538 children, 94.9% (n = 3358) had information both on the outcomes of interest and paracetamol exposure in the first trimester, and 3251 (91.9%) had information both on the outcomes of interest and paracetamol exposure in the third trimester.

Prevalence of maternal paracetamol use was 30.6% (1026 children exposed) during the first trimester and 36.7% (1194 children exposed) during the third trimester of pregnancy. The mean duration of paracetamol use was 3.3 days (standard deviation, SD 3.84 days/trimester) and 3.7 days (SD 4.55 days/trimester) in the first and third trimester respectively. The percentage of mothers reporting 5 or more days of paracetamol use was 5.5% and 6.4% in the first and third trimester respectively.

Maternal socio-demographic characteristics and potential confounders in users and non-users of paracetamol are presented in Table 1. Maternal smoking during pregnancy, presence of siblings, and maternal antibiotic use during pregnancy were more frequent among users of paracetamol than among non-users. Similarly, maternal asthma or allergies, maternal respiratory diseases and in general all maternal disorders both in the first and in the third trimester of pregnancy, were more frequent among paracetamol users than non-users (Table 1). The overall prevalence of ever wheezing in the first 18 months of life was 16.9% (567 out of 3358) and the prevalence of recurrent wheezing/asthma was 5.6% (189 out of 3358).

**Table 1:** Maternal socio-demographic characteristics and potential confounders by paracetamol use during the first and third trimesters of pregnancy. NINFEA cohort study, 2005–2013.

Maternal characteristics and potential confounders	Paracetamol use			
	First trimester		Third trimester	
	Non-users	Users	Non-users	Users
	N = 2332 (%)	N = 1026 (%)	N = 2057 (%)	N = 1194 (%)
	Missing values: -		Missing values: 107	
**Educational level**				
- University degree or higher	1423 (61.2)	582 (56.9)	1272 (62.0)	677 (57.0)
- Secondary school	789 (33.9)	389 (38.1)	688 (33.5)	446 (37.6)
- Primary school or less	112 (4.8)	51 (5.0)	92 (4.5)	64 (5.4)
- Missing values	8	4	5	7
**Age at delivery (years)**				
- <30	443 (19.0)	230 (22.4)	403 (19.6)	246 (20.6)
- 30–34	1028 (44.1)	437 (42.6)	916 (44.5)	506 (42.4)
- 35–39	712 (30.5)	301 (29.3)	605 (29.4)	371 (31.1)
- ≥40	149 (6.4)	58 (5.6)	133 (6.5)	71 (5.9)
- Missing values	0	0	0	0
**Smoking during pregnancy**				
- No	2148 (92.5)	936 (91.8)	1905 (93.0)	1086 (91.5)
- Yes	173 (7.4)	84 (8.2)	143 (7.0)	101 (8.5)
- Missing values	11	6	9	7
**Siblings**				
- No	1827 (79.2)	730 (72.3)	1651 (81.3)	824 (69.8)
- Yes	480 (20.8)	280 (27.7)	380 (18.7)	357 (30.2)
- Missing values	25	16	26	13
**Gestational duration (weeks)**				
- <37	81 (3.5)	42 (4.1)	74 (3.6)	45 (3.8)
- 37–42	2089 (89.6)	916 (89.3)	1836 (89.3)	1075 (90.0)
- ≥42	162 (6.9)	68 (6.6)	147 (7.1)	74 (6.2)
- Missing values	0	0	0	0
**Asthma/asthmatic bronchitis**				
- Never	2169 (93.0)	927 (90.3)	1915 (93.1)	1083 (90.7)
- Ever	163 (7.0)	99 (9.7)	142 (6.9)	111 (9.3)
- Missing values	0	0	0	0
**Allergic rhinitis/hay fever**				
- Never	2046 (87.7)	862 (84.0)	1808 (87.9)	1005 (84.2)
- Ever	286 (12.3)	164 (16.0)	249 (12.1)	189 (15.8)
- Missing values	0	0	0	0
**Disorders during pregnancy**				
Otitis/sinusitis/throat infection				
- No	2192 (94.5)	865 (84.3)	1992 (96.8)	1057 (88.5)
- Yes	128 (5.5)	161 (15.7)	65 (3.2)	137 (11.5)
- Missing values	12	0	0	0
Bronchitis or flu				
- No	2284 (98.4)	971 (94.6)	2017 (98.1)	1075 (90.0)
- Yes	36 (1.5)	55 (5.4)	40 (1.9)	119 (10.0)
- Missing values	12	0	0	0
Cold				
- No	2013 (86.8)	715 (69.7)	1887 (91.7)	937 (78.5)
- Yes	307 (13.2)	311 (30.3)	170 (8.3)	257 (21.5)
- Missing values	12	0	0	0
Fever >38°C				
- No	2280 (98.3)	956 (93.2)	2048 (93.9)	1097 (91.9)
- Yes	40 (1.7)	70 (6.8)	125 (6.1)	97 (8.1)
- Missing values	12	0	0	0
Sciatica				
- No	1877 (80.5)	723 (70.5)	883 (42.9)	357 (29.9)
- Yes	455 (19.5)	303 (29.5)	1174 (57.1)	837 (70.1)
- Missing values	0	0	0	0
Headache/migraine				
- No	2252 (96.6)	868 (84.8)	1965 (95.5)	957 (80.1)
- Yes	80 (3.4)	156 (15.2)	92 (4.5)	237 (19.8)
- Missing values	0	2	0	0
**Antibiotic use during pregnancy**				
- No	2100 (90.3)	873 (85.1)	1852 (90.0)	1027 (86.0)
- Yes	226 (9.7)	153 (14.9)	205 (10.0)	167 (14.0)
- Missing values	6	0	0	0

All regression models were performed on 3268 children with complete data (i.e., no missing data for any of the outcomes, exposures, or confounding variables) for analyses on the first trimester and on 3180 children for analyses on the third trimester of pregnancy.

In the crude analysis, compared to unexposed infants, the risk of ever wheezing was higher in infants exposed to paracetamol in both the first and third trimester of pregnancy (Table 2). Adjustment for the first set of potential confounders reduced the relative risks by about 9% for infants exposed in the first trimester and by 13% for those exposed in the third trimester. The relative risks further decreased after adjusting for maternal infections during pregnancy (by about 6% for exposure in the first trimester and 4% for exposure in the third trimester). A similar pattern was observed for recurrent wheezing/asthma, although analyses were based on a smaller number of events (Table 2).

**Table 2:** Association between infant wheezing at 18 months and recurrent wheezing/asthma and maternal paracetamol use in the first or third trimester. NINFEA cohort study, 2005–2013.

Outcomes	Paracetamol use				
	Number of cases	Exposed cases	RR_crude_	RR_adjusted_ ^§^	RR_adjusted_ ^§§^
	N	*(%)*	*[95% CI]*	*[95% CI]*	*[95% CI]*
**Ever wheezing**	**First trimester**				
	553	196	1.25	1.16	1.10
		*(35*.*4)*	*[1*.*07–1*.*47]*	*[1*.*00–1*.*37]*	*[0*.*94–1*.*30]*
	**Third trimester** **				
	534	225	1.26	1.13	1.10
		*(42*.*1)*	*[1*.*08–1*.*47]*	*[0*.*96–1*.*32]*	*[0*.*93–1*.*29]*
**Recurrent wheezing/asthma**	**First trimester**				
	185	67	1.30	1.15	1.10
		*(36*.*2)*	*[0*.*97–1*.*73]*	*[0*.*86–1*.*53]*	*[0*.*82–1*.*49]*
	**Third trimester** **				
	178	68	1.07	0.89	0.81
		*(38*.*2)*	*[0*.*80–1*.*43]*	*[0*.*66–1*.*20]*	*[0*.*60–1*.*11]*

^§^ Adjusted for: maternal educational level, maternal age at delivery, maternal smoking during pregnancy, siblings, maternal asthma/asthmatic bronchitis, maternal allergic rhinitis.

^§§^ Adjusted analysis for the same variables as footnote ^§^, as well as for maternal infections (or factors associated with maternal infections) in the first/third trimester of pregnancy: otitis/sinusitis/throat infections, bronchitis or flu, cold, fever, and antibiotic use during pregnancy.

**Adjusted further for gestational duration.

RR: risk ratio, CI: confidence interval.

In adjusted analyses, duration of paracetamol use during the first trimester as well as the third trimester of pregnancy was not associated with an increased risk of recurrent wheezing/asthma in the first 18 months of life (first trimester: RR_adjusted_ = 0.99 [95% CI 0.95–1.02] for ever wheezing and RR_adjusted_ = 0.96 [95% CI 0.89–1.03] for recurrent wheezing/asthma; third trimester: RR_adjusted_ = 1.00 [95% CI 0.98–1.02] for ever wheezing, RR_adjusted_ = 0.99 [95% CI 0.94–1.04] for recurrent wheezing/asthma; data not shown). When analyses on duration of paracetamol use were carried out using spline regression to accommodate possible non-linear relationships, the lack of association between duration of paracetamol use and wheezing was confirmed.

Table 3 shows the results on paracetamol use for selected non-infectious disorders (sciatica, headache, and migraine), separately in the first and in third trimester of pregnancy. Although this analysis is based on relatively small numbers, we did not find evidence of an increased risk of wheezing or asthma in the first 18 months of life among infants whose mothers took paracetamol to treat these non-infectious disorders compared to mothers that did not.

**Table 3:** Risk ratios (RR), and corresponding 95% confidence Intervals (CI), of ever wheezing and recurrent wheezing/asthma for maternal paracetamol use to treat sciatica or migraine or headache in the first and third trimester of pregnancy. NINFEA cohort study, 2005–2013.

	**Paracetamol use in the first trimester**		
	Exposed cases	RR_crude_	RR_adjusted_ ^§^
	*(%)*	*[95% CI]*	*[95% CI]*
**Ever wheezing**			
Women affected by sciatica, migraine or headache and NOT using paracetamol to treat them	77	1.00	1.00
	*(82*.*8)*	*ref*	*ref*
Women affected by sciatica, migraine or headache and using paracetamol to treat them	16	1.04	1.06
	*(17*.*2)*	*[0*.*64–1*.*70]*	*[0*.*65–1*.*72]*
**Recurrent wheezing/asthma**			
Women affected by sciatica, migraine or headache and NOT using paracetamol to treat them	28	1.00	1.00
	*(84*.*8)*	*ref*	*ref*
Women affected by sciatica, migraine or headache and using paracetamol to treat them	5	0.90	0.91
	*(15*.*2)*	*[0*.*36–2*.*26]*	*[0*.*38–2*.*20]*
	**Paracetamol use in the third trimester** *		
	Exposed cases	RR_crude_	RR_adjusted_ ^§^
	*(%)*	*[95% CI]*	*[95% CI]*
**Ever wheezing**			
Women affected by sciatica, migraine or headache and NOT using paracetamol to treat them	106	1.00	1.00
	*(74*.*6)*	*ref*	*ref*
Women affected by sciatica, migraine or headache and using paracetamol to treat them	36	0.76	0.70
	*(25*.*4)*	*[0*.*54–1*.*07]*	*[0*.*50–0*.*97]*
**Recurrent wheezing/asthma**			
Women affected by sciatica, migraine or headache and NOT using paracetamol to treat them	39	1.00	1.00
	*(84*.*8)*	*ref*	*ref*
Women affected by sciatica, migraine or headache and using paracetamol to treat them	7	0.40	0.34
	*(15*.*2)*	*[0*.*18–0*.*88]*	*[0*.*15–0*.*74]*

^§^ Adjusted analysis for: maternal educational level, maternal age at delivery, smoking in pregnancy, siblings, maternal asthma or asthmatic bronchitis, maternal allergic rhinitis.

* Further adjusted for gestational duration.

S4 Table reports the results of the associations (expressed as odds ratios) between maternal conditions and paracetamol use by trimester, calculated for the propensity score-based analysis. The propensity score itself was associated with the risk of ever and recurrent wheezing, with RRs of 1.18 [95% CI 0.82–1.69] and 1.21 [95% CI 0.56–2.59] in the first trimester and RRs of 3.37 [95% CI 2.04–5.54] and 4.18 [95% CI 1.35–12.95] in the third trimester for each 1-unit increase. When the propensity score was included in the regression models on paracetamol use and risk of wheezing in infants, we obtained results similar to those in the main analysis reported in Table 2 (first trimester: RR_adjusted_ = 1.11 [95% CI 0.89–1.37] for ever wheezing and RR_adjusted_ = 1.10 [95% CI 0.72–1.67] for recurrent wheezing/asthma; third trimester: RR_adjusted_ = 1.05 [95% CI 0.85–1.30] for ever wheezing and RR_adjusted_ = 0.89 [95% CI 0.58–1.337] recurrent wheezing/asthma; data not shown in tables).

## Discussion

We found a crude positive association between prenatal exposure to paracetamol and ever wheezing and recurrent wheezing/asthma in infants. However, adjustment for confounders that have been identified in studies on childhood wheezing and for maternal infections and factors associated with maternal infections during pregnancy substantially decreased the relative risk estimates, even when a propensity score-based analysis was applied. Moreover, there was no evidence of an association between duration of paracetamol use and the risk of wheezing in children. Finally, we did not find an increased risk for maternal paracetamol use to treat non-infectious disorders (sciatica and headache/migraine) and wheezing in infants. These results suggest that confounding is a likely explanation for at least most of the association between paracetamol use in pregnancy and wheezing in children.

Several cohort studies reported a positive association between prenatal exposure to paracetamol and wheezing or asthma in children (summarised in S5 and S6 Tables), but the lack of complete adjustment for factors that could both indicate paracetamol use in pregnancy and that are related to wheezing/asthma in children, was typically mentioned as a possible explanation for the positive findings [3, 5, 8, 9, 22–24]. Several factors can be proxies of paracetamol use during pregnancy, and at the same time may be related to wheezing or asthma in children (e.g. maternal asthma and allergic rhinitis). In addition, environmental factors that favour maternal infections (e.g. maternal smoking, low socio-economic status, or parity) may affect maternal paracetamol use and increase the risk of infections in infancy, which in turn may trigger wheezing. In our study, we observed a large reduction in the estimates of the association between maternal paracetamol use and wheezing in infancy when we adjusted for these environmental factors and maternal asthma and allergies. Furthermore, we also had direct information on maternal respiratory infections and associated variables (use of antibiotics, cold), and adjustment for these factors further reduced our estimates towards unity.

Most of the previous studies adjusted for several potential confounders (listed in S5 and S6 Tables), and in general these adjustments consistently attenuated the positive estimates. However, a sizable and heterogeneous number of variables were usually adjusted for, making it difficult to understand the relative contribution of each confounder. Furthermore, among the studies that examined the association between maternal paracetamol use and wheezing/asthma in children (S5 and S6 Tables), very few were able to take into account maternal infections, particularly maternal respiratory infections. Shaheen et al. adjusted for some maternal infections (cold/flu, urinary infections, other infections) and antibiotic use [22, 25], but only those that occurred in late pregnancy; four studies considered only antibiotic use in pregnancy [9, 26, 27], or in late pregnancy [23]. Our study shows that maternal infections during pregnancy contribute to confounding bias, and suggests that the lack of adequate control for this group of confounders could at least partly explain the large between-study heterogeneity that has been previously observed [3].

In an attempt to analyse the effect of maternal paracetamol use while avoiding the problem of confounding due to maternal infections, we used a novel approach: we analysed maternal paracetamol use for non-infectious disorders, including migraine, headache, and sciatica. Results were still consistent, indicating no increased risk of wheezing in children for maternal paracetamol use during pregnancy.

After adjustment, we did not find an association between wheezing and maternal paracetamol use during either the first or third trimester of pregnancy. Had the effect been causal, the timing of exposure to paracetamol use during pregnancy could have been related to biological mechanisms [27, 28]. We did not ascertain paracetamol use in the second trimester of pregnancy, and thus we could not assess the effect of exposure throughout the entire pregnancy. Available studies on timing of paracetamol exposure during pregnancy have reported conflicting results (S5 and S6 Tables): some studies found an increased risk for exposure to paracetamol only in late pregnancy [23, 25]; others for exposure in both early and late pregnancy [12, 24, 27]; and still others, like our study, found no association in either early or late pregnancy [11].

As in other studies [13, 23, 25, 26], we focused our analysis on wheezing, or asthma diagnosed by a physician occurring in the first 18 months of life rather than on asthma at older ages, given the relatively young age of our cohort. The use of asthmatic symptoms reported by the mother may involve a certain degree of misclassification; however, such misclassification should not have been influenced by prenatal exposure to paracetamol, which is very commonly used and is not directly linked with a fear of harmful effects on the respiratory health of children. Since parents might wrongly label a single, isolated episode of noisy breathing as wheezing [29], and since wheezing in infants can be a transient disorder, we also considered infants with recurrent wheezing (i.e. at least two episodes of wheezing by the age of 18 months), a more specific condition that reflects “actual” wheezing episodes or a more severe form of wheezing, and also we obtained comparable results.

In our study, information on maternal paracetamol use and disorders during the first trimester of pregnancy was collected at any time during the pregnancy, while information on the third trimester was collected in the first follow-up questionnaire when the child turned 6 months old. As maternal paracetamol use was self-reported, it could be affected by some degree of misclassification. However, this is expected to be non-differential, especially for the first trimester, and likely biased the estimates towards the null. On the other hand, it could be underestimated due to exposure misclassification. It is reassuring that when we studied paracetamol use to treat sciatica and headache/migraine, this exposure was not associated with the risk of wheezing in children. The level of exposure misclassification in this analysis is likely to be lower.

Our study was based on a relatively large cohort with baseline information on several recognised risk factors for wheezing and asthma, including maternal educational level, smoking, and various maternal infectious diseases and disorders during pregnancy. We specifically focused on the role of confounding, adjusting for a large number of potential confounders and conducting further analyses, including a propensity score-based analysis, to validate our results. However, other factors not considered in the present analysis, e.g. maternal stress/anxiety [8], could play a role in the association between maternal paracetamol use in pregnancy and childhood asthma, and deserve future investigation.

In conclusion, our results support the hypothesis that the repeatedly reported association between maternal paracetamol use during pregnancy and infant wheezing can be mainly, if not completely explained by confounding.

## Supporting Information

S1 Table Original questions from the NINFEA cohort study questionnaire used to determine maternal paracetamol use. (DOC)

S2 Table Original questions from the NINFEA cohort study questionnaire used to determine maternal paracetamol use for selected non-infective disorders. (DOC)

S3 Table Original questions on wheezing from the NINFEA cohort study questionnaire. (DOC)

S4 Table Associations between maternal disorders and paracetamol use by trimester, using a propensity score-based analysis. NINFEA cohort study, 2005–2013. (DOC)

S5 Table Birth cohort studies on the association between maternal paracetamol use and paediatric wheezing or asthma. (DOC)

S6 Table Registry-based cohort studies on the association between pre-natal paracetamol exposure and paediatric wheezing or asthma. (DOC)
