# Supplementary material for: Prenatal Paracetamol Exposure and Wheezing in Childhood: Causation or Confounding?
Source: PLoS One. 2015 Aug 25;10(8):e0135775. doi: 10.1371/journal.pone.0135775 (PMC4549146; doi:10.1371/journal.pone.0135775)
Supplement: S1 Table — (DOC) [file pone.0135775.s001.doc]

**S1 Table. Original questions from the NINFEA cohort study questionnaire used to determine maternal paracetamol use.**

| **Exposure** | **Questionnaire filled in** | **Questions** |
| --- | --- | --- |
| Paracetamol use in the first/ third trimester | during pregnancy | Have you taken paracetamol during the pregnancy or three months before pregnancy? [*No; Yes*] |
|  |  | Have you taken paracetamol in the first, second and/or third month of pregnancy? [*No; Yes*] |
|  | 6 months after delivery | Have you taken paracetamol during the third trimester of pregnancy? [*No; Yes*] |
| Dose of paracetamol in the first / third trimester | during pregnancy | How many days have you taken paracetamol in the first, second and/or third month of pregnancy? *[1-2 days; 3-7 days; more than 7 days]* [Number of days]*** |
|  | 6 months after delivery | How many days have you taken paracetamol during the third trimester of pregnancy?  *[1-2 days; 3-7 days; more than 7 days]* [Number of days]*** |

* Questionnaire valid from 2005 to 2007

** Questionnaire valid from 2008 to present
