# Supplementary material for: Prenatal Paracetamol Exposure and Wheezing in Childhood: Causation or Confounding?
Source: PLoS One. 2015 Aug 25;10(8):e0135775. doi: 10.1371/journal.pone.0135775 (PMC4549146; doi:10.1371/journal.pone.0135775)
Supplement: S2 Table — (DOC) [file pone.0135775.s002.doc]

**S2 Table. Original questions from the NINFEA cohort study questionnaire used to determine maternal paracetamol use for selected non-infective disorders.**

| **Exposure** | **Questionnaire filled in** | **Questions** |
| --- | --- | --- |
| Paracetamol taken to treat selected non-infective disorders in the first/third trimester | During pregnancy | Have you been diagnosed with *sciatica* (*migraine, headache*) by a doctor? [*No; Yes*]  When have you had recurrent *sciatica* (*migraine, headache*)? [*Before pregnancy; During pregnancy; Both before and during pregnancy*] |
|  |  | Have you taken medication to treat *sciatica* (*migraine, headache*)? [*No - never; Yes – three months before pregnancy; Yes – also (or only) during pregnancy; Yes – but not during pregnancy*]  Indicate the commercial or generic name of medication you have been taking to treat *sciatica* (*migraine, headache*) during pregnancy. [*Name of medication*]  How many days have you been taking this medication in the first [*second; third*] month of pregnancy?  [*0 days; 1-2 days; 3-7 days; more than 7 days*] |
|  | 6 months after delivery | Have you had *sciatica* (*migraine, headache*) during the third trimester of pregnancy? [*No; Yes*] |
|  |  | Have you taken medication to treat *sciatica* (*migraine, headache*) during the third trimester of pregnancy? [*No; Yes*] |
|  |  | Indicate the commercial or generic name of the medication you have been taking to treat *sciatica* (*migraine, headache*) during the third trimester of pregnancy. [*Name of medication*] |
