# Supplementary material for: Prenatal Paracetamol Exposure and Wheezing in Childhood: Causation or Confounding?
Source: PLoS One. 2015 Aug 25;10(8):e0135775. doi: 10.1371/journal.pone.0135775 (PMC4549146; doi:10.1371/journal.pone.0135775)
Supplement: S3 Table — (DOC) [file pone.0135775.s003.doc]

**S3 Table. Original questions on wheezing from the NINFEA cohort study questionnaire.**

| **Outcomes** | **Questionnaire filled in** | **Questions** |
| --- | --- | --- |
| Ever wheezing | 6 months after delivery | Has your child had episodes of wheezing or whistling in the chest in the first 6 months of life? |
|  | 18 months after delivery | Has your child had episodes of wheezing or whistling in the chest from 6 to 18 months of life? |
| Asthma diagnosis | 6 months after delivery | Has your child been diagnosed with asthma by a doctor in the first 6 months of life? |
|  | 18 months after delivery | Has your child been diagnosed with asthmatic bronchitis or bronchial asthma by a doctor between 6 and 18 months of life? |
