# Supplementary material for: Prenatal Paracetamol Exposure and Wheezing in Childhood: Causation or Confounding?
Source: PLoS One. 2015 Aug 25;10(8):e0135775. doi: 10.1371/journal.pone.0135775 (PMC4549146; doi:10.1371/journal.pone.0135775)
Supplement: S4 Table — (DOC) [file pone.0135775.s004.doc]

**S4 Table. Associations between maternal disorders and paracetamol use by trimester, using a propensity score-based analysis. NINFEA cohort s**tudy, 2005-2013.

|  | **Paracetamol use in the first trimester of pregnancy** | | **Paracetamol use in the third trimester of pregnancy** | |
| --- | --- | --- | --- | --- |
|  | **Mutually adjusted OR** | **[95% CI]** | **Mutually adjusted OR** | **[95% CI]** |
| ***Maternal asthma or allergies*** |  |  |  |  |
| - Maternal asthma/asthmatic bronchitis (ever) | 1.15 | [0.96, 1.37] | 1.11 | [0.94, 1.32] |
| - Maternal allergic rhinitis/hay fever (ever) | 1.11 | [0.97, 1.28] | 1.16 | [1.01, 1.33] |
| ***Maternal respiratory disorders in the first trimester of pregnancy (yes)*** |  |  |  |  |
| - Otitis/sinusitis/throat infection | 1.48 | [1.25, 1.75] | 1.16 | [0.98, 1.37] |
| - Bronchitis or flu | 1.47 | [1.12, 1.96] | 1.00 | [0.75, 1.33] |
| - Cold | 1.65 | [1.46, 1.86] | 1.10 | [0.98, 1.25] |
| - Fever >38°C | 1.82 | [1.40, 2.36] | 1.37 | [1.06, 1.76] |
| ***Antibiotic use*** |  |  |  |  |
| - Yes | 1.25 | [1.08, 1.44] | 1.21 | [1.05, 1.39] |

OR: odds ratios, CI: confidence interval.
