# Supplementary material for: Prenatal Paracetamol Exposure and Wheezing in Childhood: Causation or Confounding?
Source: PLoS One. 2015 Aug 25;10(8):e0135775. doi: 10.1371/journal.pone.0135775 (PMC4549146; doi:10.1371/journal.pone.0135775)
Supplement: S5 Table — (DOC) [file pone.0135775.s005.doc]

**S5 Table. Birth cohort studies on the association between maternal paracetamol use and paediatric wheezing or asthma.**

| **Country (Author)** | **Study population** | **Outcome** | **Exposure** | **Associations RR/OR (95% CI)^a^** | **Confounders** |
| --- | --- | --- | --- | --- | --- |
| UK (Shaheen SO, 2002) | 13,988 live births  (1991-1992, ALSPAC cohort) | Wheezing  (30-42months) | 0-20 weeks of pregnancy  20-32 weeks of pregnancy | 1.19 (0.72-1.97) for frequent users (most days/daily)  2.10 (1.30-3.41) for frequent users (most days/daily | Child’s sex, mother’s education level, mother’s housing tenure, mother’s ethnic origin, parity, mother’s age, birth season, breastfeeding in first 6 months, day care use in first 8 months, antibiotic use in first 6 months, pets kept in first year, maternal smoking in pregnancy, weekend tobacco exposure in first 6 months, mother’s BMI, crowding in home, damp and/or mould in home, child’s birthweight, child’s gestational age at birth, multiple pregnancy, child’s head circumference, child’s birth length.  *Maternal diseases:* Ever asthma, ever eczema, ever rhinitis, ever migraine, history of atopic/migraine, cold/flu in late gestation, urinary infection in late gestation, other infections in late gestation, mother’s history of infections unknown mother’s anxiety score in pregnancy. |
| UK (Shaheen SO, 2005) | 8411 children aged 7 years (ALSPAC cohort) | Wheezing  (69-81 months)  ------------------  Asthma  (69-81 months) | 0-20 weeks of pregnancy  20-32 weeks of pregnancy  -----------------  0-20 weeks of pregnancy  20-32 weeks of pregnancy | 1.81 (1.05-3.13)  for frequent users (most days/daily)  1.86 (0.98-3.55)  for frequent users (most days/daily)  -----------------------  1.29 (0.74-2.27)  for frequent users (most days/daily)  1.62 (0.86-3.04)  for frequent users (most days/daily) | *Primary confounders:* Child’s sex, child’s birthweight, Child’s gestational age at birth, child’s head circumference, mother’s age, maternal smoking in pregnancy, mother’s education level, mother’s housing tenure, mother’s parity, mother’s anxiety score in pregnancy, mother’s ethnic origin, multiple pregnancy, mother ever had asthma, eczema, rhinitis, migraine, history of these conditions unknown. Mother had cold/flu in late pregnancy, mother had urinary infection in late pregnancy, mother had other infections in late pregnancy, mother’s history of infections unknown, maternal antibiotic use in late pregnancy.  *Secondary confounders:* Number of younger siblings at 7 years, pets in first year, breastfed in first 6 months, day care use in first 8 months, damp and/or mould in home, weekend tobacco exposure in first 6 months, child’s body mass index at 7 years. |
| Denmark (Rebordosa C, 2008) | DNBC cohort  66,445 (1996-2003)  12,733 (1996-1999) | Wheezing ever (at 18 months)  Wheezing in the last year (at 7-years) | Overall exposure during pregnancy | 1.13 (1.10-1.17)  1.16 (0.99-1.35) | Mother's asthma, child's sex, socioeconomic status, gestational age at birth, duration of breastfeeding, smoking during pregnancy, and antibiotic use during pregnancy. *Other potential confounders evaluated (but not included in the analyses- the authors report associations unchanged after control for):* Mother’s age, birth order, mother had fever during pregnancy, mother had muscle or joint disease during pregnancy, mother had inflammation or infection during pregnancy, mother had hypertension during pregnancy, mother ever had allergy, mother ever had eczema, pets during the first 18 months of life, alcohol consumption during pregnancy. |
| **Country (Author)** | **Study Population** | **Outcome** | **Exposure** | **Associations RR/OR (95% CI) ^a^** | **Confounders** |
| USA (Persky, 2008) | 345 pregnant women | Wheezing (1^st^ year of life) | During pregnancy  Early pregnancy Middle-late pregnancy | 1.7 (1.0-3.0)  1.0 (0.6-1.6) 1.8 (1.1-3.0) | Maternal age, child's sex, home environment intervention group, maternal Mexican ethnicity, child breastfed for 4 or more weeks, active smoking in middle to late pregnancy, exposure to passive smoke during pregnancy, low birth weight, antibiotic use in late pregnancy, age at which formula introduced, and family history of asthma. *Other potential confounders evaluated (data not shown, the authors report associations unchanged after control for):* upper respiratory tract infections (cough, cold, or sore throat) at any time during pregnancy, maternal education, number of previous live births (parity), acetaminophen use in the first year of life, gestational age younger than 36 weeks, measures of exposure to passive smoke in early, middle, or late pregnancy, and low antioxidant intake in pregnancy (for a subset of 312 women with complete dietary data). |
| USA (Perzanowsky, 2009) | 301 children | Current wheezing  (at 5 years) | During pregnancy  First trimester Second trimester Third trimester | 1.71 (1.20-2.43)  1.41 (0.93-2.14) 1.54 (1.06-2.25) 1.55 (1.03-2.34) | Sex, ethnicity, birth order, maternal asthma, maternal hardship, ETS exposure and postnatal acetaminophen use. |
| USA (Kang, 2009) | 1,505 pregnant women | Ever asthma (during 6 years of life) | During pregnancy  First trimester Third trimester | 0.76 (0.53-1.10)  0.68 (0.39-1.20) 0.91 (0.48-1.69) | Yearly household income, household exposures (mold/mildew growth at home and water leaks/damage at home during child’s first year, pets inside home and cockroaches observed in home during child’s first and sixth years), use of various home appliances, father’s ethnicity and education, child’s ethnicity, siblings, attendance at a programme before elementary school, breastfeeding, mother’s use of antibiotics while breastfeeding, child’s exposure to tobacco smoke for 2 hours or more ever.  ------------------------------------------  *Child's conditions and diseases:* Asthma symptoms (wheezing and persistent cough in the first year, cough, shortness of breath, and chest tightness in the sixth year), use of emergency department and overnight stay at the hospital for asthma, allergy, or respiratory illnesses, use of neonatal intensive care unit and paediatric intensive care unit, use of intubation/ventilation in neonatal intensive care unit and paediatric intensive care unit, child’s use of antibiotics and allergy medications, and child’s infections and respiratory illnesses (allergies, sneezing/runny nose, hay fever, itchy rash, eczema, bronchitis, bronchiolitis, pneumonia, croup, ear infection, strep throat, sinus infection, respiratory syncytial virus, tonsillitis). *Maternal/Paternal diseases:* Mother’s diagnosed or treated eczema, history of father’s asthma and other health conditions (wheezing, allergies, and eczema). |
| **Country (Author)** | **Study Population** | **Outcome** | **Exposure** | **Associations RR/OR (95% CI) ^a^** | **Confounders** |
| Sweden (Goksor 2011) | 4496 children | Recurrent wheezing  Inhaled-corticosteroid treated wheezing | During pregnancy | - 1. (0.6-2.0)   1.6 (1.01-2.6) | Male gender, maternal smoking during pregnancy, gestational age <37 weeks, caesarean section, asphyxia (Apgar at 5 min <7), treatment with broad-spectrum antibiotics during the first week of life, breast-feeding for 4 months or more, early fish introduction, own eczema or doctor-diagnosed food allergy during the first year of life and parental level of education.  Having a mother or father with asthma, eczema or rhinoconjunctivitis, maternal antibiotic use during pregnancy. |
| Norway (Bakkeheim 2011) | 1019 children | Current asthma (history of asthma plus symptoms or medication within the last  year or a positive exercise test) at 10 years | First trimester  Second/third trimester | 1.04 (0.39 - 2.75)  1.0 (0.38 , 2.62) | Crude results.  *Note:*  for the secondary outcome “HISTORY OF ASTHMA” (=at least two of three criteria: 1. Dyspnoea, chest tightness and ⁄ or wheezing. 2. Doctor’s diagnosis of asthma. 3. Use of asthma medication) the following Crude OR were estimated  First trimester: RR 1.51 (0.72-3.14)  Second/Third trimester: RR 1.43 (0.69-2.96) |
| Denmark (Kreiner-Moller,  2012) | 411 children born to asthmatic mothers | Troublesome lower lung symptoms  (0-1 year)  Current asthma (at 7 year) | Third trimester of pregnancy | 0.98 (0.73-1.31)  0.95 (0.66-1.37) | Crude results. |
| US (Sordillo, 2015) | 1490 pregnant women | Recurrent wheezing  (3-5 years)  Asthma  (3-5 years)  Persistent wheezing (7-10 years)  Current asthma  (7-10 years) | During pregnancy | 1.41 (1.06-1.89)  1.26 (1.02-1.58)   - 1. (0.66-1.60)   1.25 (0.94-1.65) | Four analgesic exposures (acetaminophen in infancy, ibuprofen in infancy, prenatal acetaminophen, and prenatal ibuprofen), child’s sex and multivitamin intake, mother’s age at enrolment, race/ethnicity, prepregnancy BMI, household income, number of children less than 12 years of age in the home, breastfeeding duration, passive smoking exposure, smoking during pregnancy, child care attendance, maternal and paternal history of asthma. |

| **Country (Author)** | **Study Population** | **Outcome** | **Exposure** | **Associations RR/OR (95% CI) ^a^** | **Confounders** |
| --- | --- | --- | --- | --- | --- |
| Italy (this study, 2015) | 3,538 singleton pregnancies | Ever wheezing in the first 18 months  Recurrent wheezing in the first 18 months or asthma diagnosis | First trimester of pregnancy  Third trimester of pregnancy  First trimester of pregnancy    Third trimester of pregnancy | 1.10 (0.93-1.30)  1.09 (0.93-1.28)  1.12 (0.83-1.52)  0.81 (0.59-1.11) | Maternal educational level, smoking in pregnancy, siblings, maternal age at birth, maternal asthma or asthmatic bronchitis, maternal allergic rhinitis, and maternal infections in the first/third trimester: bronchitis or flu, otitis or sinusitis and throat infections, fever, cold, and antibiotic use during pregnancy. |

**^a^** RR: risk ratio, OR: odds ratio, CI: confidence interval.
