# Supplementary material for: Prenatal Paracetamol Exposure and Wheezing in Childhood: Causation or Confounding?
Source: PLoS One. 2015 Aug 25;10(8):e0135775. doi: 10.1371/journal.pone.0135775 (PMC4549146; doi:10.1371/journal.pone.0135775)
Supplement: S6 Table — (DOC) [file pone.0135775.s006.doc]

**S6 Table. Registry-based cohort studies on the association between pre-natal paracetamol exposure and paediatric wheezing or asthma.**

| **Country (Author)** | **Study Population** | **Outcome** | **Exposure** | **Associations**  **RR/OR (95% CI)^a^** | **Confounders** |
| --- | --- | --- | --- | --- | --- |
| Denmark (Andersen, 2012) | 197,060 children | Asthma incidence (hospitalization, outpatient visit, or emergency room visit with a diagnosis of asthma or a dispensation record for anti-asthma medication) | Dispensation during pregnancy  First trimester  Second/third trimester | 1.40 (1.16–1.71)  1.28 (1.02–1.62) | Year of birth, county of residence, child’s sex, gestational age, birth order, delivery mode, mother’s age at delivery, maternal smoking during pregnancy, maternal use of systemic antibiotics during pregnancy, maternal asthma, maternal prepregnancy body mass index |
| Sweden  (Kallen 2013) | 685,015  Children  571,277  Children after exclusion of mothers who received anti-asthmatic drugs | Asthma (at least five separate prescriptions of drugs) | Prescription  second/third trimester | 1.50 (1.37–1.63)  After exclusion of mothers who received anti-asthmatic drugs :  1.13 (0.95–1.31) | Year of birth, maternal age, parity, smoking, and body mass index |

**^a^** RR: risk ratio, OR: odds ratio, CI: confidence interval.
